# Allelopathy and its application as a weed management tool: A review

**DOI:** 10.3389/fpls.2022.1034649

**Published:** 2022-11-28

**Authors:** Yuvraj Khamare, Jianjun Chen, Stephen Christopher Marble

**Affiliations:** Environmental Horticulture Department, Mid-Florida Research and Education Center, Institute of Food and Agricultural Sciences, University of Florida, Apopka, FL, United States

**Keywords:** allelochemicals, natural herbicides, bioherbicides, weed control, intercropping

## Abstract

Weeds are a serious threat to crop production as they interfere with the crop growth and development and result in significant crop losses. Weeds actually cause yield loss higher than any other pest in crop production. As a result, synthetic herbicides have been widely used for weed management. Heavy usage of synthetic herbicides, however, has resulted in public concerns over the impact of herbicides on human health and the environment. Due to various environmental and health issues associated with synthetic herbicides, researchers have been exploring alternative environmentally friendly means of controlling weed. Among them, incorporating allelopathy as a tool in an integrated weed management plan could meaningfully bring down herbicide application. Allelopathy is a biological phenomenon of chemical interaction between plants, and this phenomenon has great potential to be used as an effective and environmentally friendly tool for weed management in field crops. In field crops, allelopathy can be applied through intercropping, crop rotation, cover crops, mulching and allelopathic water extracts to manage weeds. Accumulating evidence indicates that some plant species possess potent allelochemicals that have great potential to be the ecofriendly natural herbicides. This review is intended to provide an overview of several allelopathic species that release some form of the potent allelochemical with the potential of being used in conventional or organic agriculture. Further, the review also highlights potential ways allelopathy could be utilized in conventional or organic agriculture and identify future research needs and prospects. It is anticipated that the phenomenon of allelopathy will be further explored as a weed management tool, and it can be a part of a sustainable, ecological, and integrated weed management system.

## Introduction

Weeds are one of the most challenging problems facing agricultural production all over the world. Weeds compete for light, nutrients, water, and space that reduces crop growth and yield. Additionally, weeds also harbor insect pests, bacterial, fungal, and virual pathogens, further reducing the crop yield. With global population expected to reach over 9 billion by 2050, world food production cannot risk any significant yield loss due to weed competition (Chauhan, 2020). Agriculture in developed countries mainly rely on synthetic herbicides to control weeds. However, herbicides with no new mechanism of action (MOA) have been introduced since the 1980s. The heavy reliance on herbicides with similar MOA has resulted in 513 unique cases of herbicide- resistant weeds globally across 267 species (Heap, 2022). The United States (U.S.) has the highest number of cases of herbicide resistance with more than 160 species. In addition, there are several other negative consequences related to heavy use of herbicides, such as high chemical costs, potential leaching and runoff into groundwater, or concerns with recycling irrigation water (Poudyal and Cregg, 2019). Public concerns over the impact of herbicides on human health and the environment are also increasing. Due to the evolution of herbicide-resistant weeds, lack of new herbicides with new MOA, and public awareness with synthetic herbicides, there is a need to develop a sustainable ecofriendly tool to manage weeds. One great field for discovering such tools is the use of plant based natural compounds called allelochemicals to control weeds. These allelopathic chemicals have phytotoxic activities with the potential to be used for suppressing certain weeds as natural herbicides. The focus of this review is to provide an overview of past research on allelopathic species and use of allelopathy in crop production, highlight potential ways allelopathy could be utilized, and identify future research needs and prospects. Examples of different allelopathic species with the potential for use in conventional or organic agriculture are also presented.

## Allelopathy and allelochemicals

Allelopathy refers to the direct or indirect effect of plants upon neighboring plants or their associated microflora or microfauna by the production of allelochemicals that interfere with the growth of the plant (IAS, 2018). The allelochemicals released from the plants act as a defense system against microbial attack, herbivore predation, or competition from other plants (Kong et al., 2019). The study of allelopathy is a sub-discipline of chemical ecology that focuses on the effects of chemicals produced by plants or microorganisms on the growth and development of other plants in natural or agricultural systems (Einhellig, 1995). The effect can be either positive or negative on the growth of the surrounding plants. The word allelopathy is derived from two separate Greek words, *allelon* meaning of each other or mutual and *pathos* meaning to suffer or feeling. Even though the term ‘allelopathie’ was first used by Austrian scientist Hans Molisch in 1937 (Willis, 2007), the chemical interaction between plants has been known for thousands of years. In 300 B.C, the Greek botanist Theophrastus mentioned the negative effects of chickpea on other plants and later Pliny, a Roman scholar (1 A.D.) noted the inhibitory effect of the walnut tree (*Juglans* spp.) over nearby crops. The allelochemicals are released from plant parts by leaching from leaves or litter on the ground, root exudation, volatilization from leaves, residue decomposition, and other processes in the natural and agricultural systems (Rice, 1984; Anaya, 1999). Upon release, the allelochemicals can suppress the germination, growth, and establishment of the surrounding plants or modify the soil properties in the rhizosphere by influencing the microbial community (Weir et al., 2004; Zhou et al., 2013). Since allelopathic substances play an important role in regulating the plant communities, they can also be used as natural biodegradable herbicides (Duke S. et al., 2000; Vyvyan, 2002).

**Figure 1:**
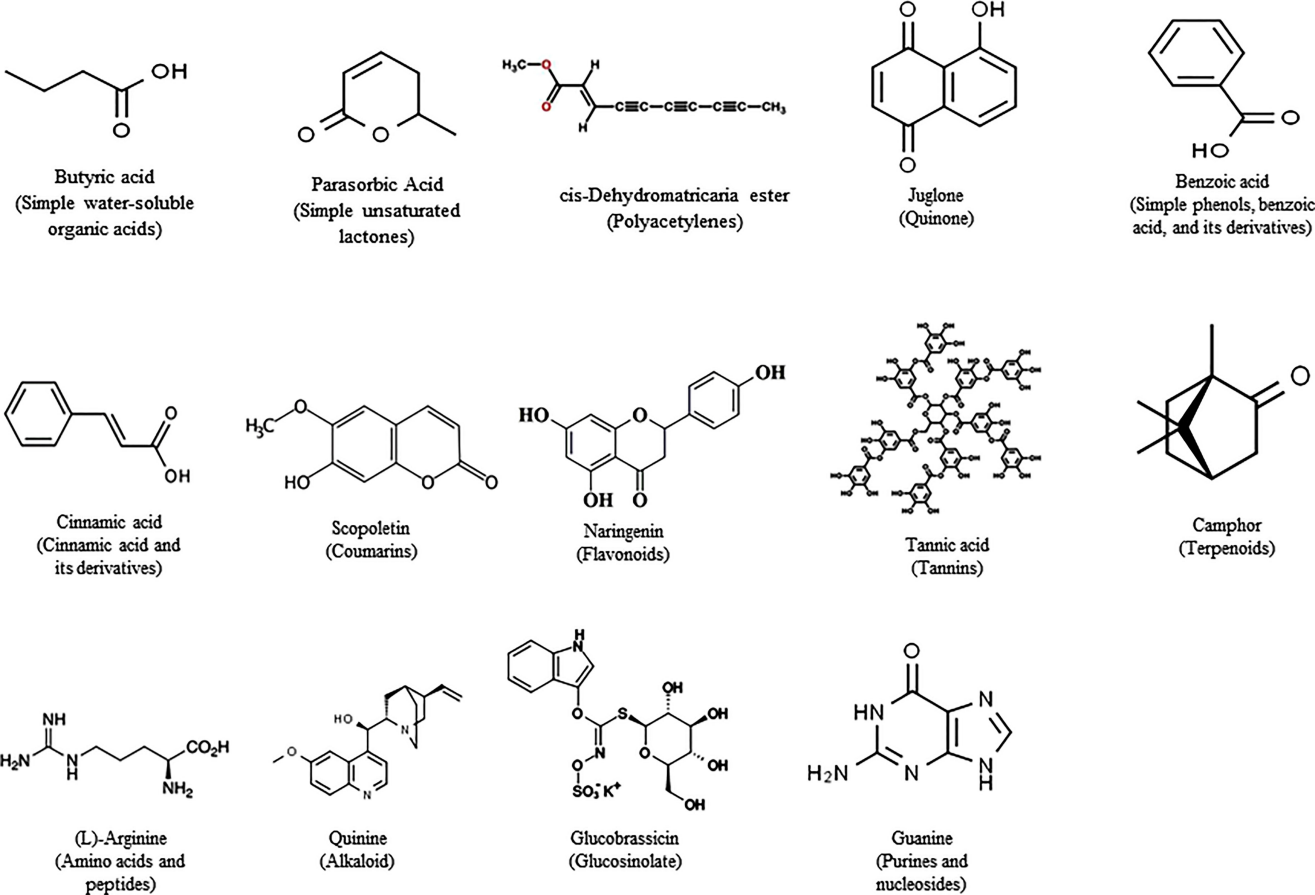
Representive allelochemicals from the classified 14 categories based on their chemical similarities.

Research on allelopathy was traditionally focused on assessing the phytotoxic activities of plant residues or crude extracts (Weston, 1996). Recent allelopathic studies made great improvements with rapid progress in separation and structural elucidation techniques, active compounds can be detected, isolated, and characterized (Mallik, 2000; Scavo et al., 2019). Allelochemicals are produced by plants as secondary metabolites or by microbes through decomposition. Allelochemicals are classified into 14 categories based on their chemical similarities (Rice, 1984). The 14 categories are water-soluble organic acids, straight-chain alcohols, aliphatic aldehydes and ketones; simple unsaturated lactones; long-chain fatty acids and polyacetylenes; benzoquinone, anthraquinone and complex quinones; simple phenols, benzoic acid and its derivatives; cinnamic acid and its derivatives; coumarin; flavonoids; tannins; terpenoids and steroids; amino acids and peptides; alkaloids and cyanohydrins; sulfide and glucosinolates; and purines and nucleosides (Cheng and Cheng, 2015) (**Figure 1**). Plant growth regulators, such as salicylic acid, gibberellic acid, and ethylene are also considered to be allelochemicals. Allelochemicals vary in mode of action, uptake, and effectiveness (Weston and Duke, 2003; Rice, 2012). The mode of action for many of the identified allelochemicals is still unclear. Many allelochemicals have mechanisms that are not used by any of the synthetic herbicides, giving researchers leads to new mode of action (Duke et al., 2002). While the efficacy and specificity of many allelochemicals are unknown or limited (Bhadoria, 2010), they are an appropriate alternative for synthetic herbicides. Many plant species have been listed worldwide for their allelopathic effects, and of these, we have listed a few with great potential for further research.

## Plants with allelopathic potential for weed control

### Black walnut (*Juglans nigra* L.)

One of the oldest and most well-researched allelopathic species is the walnut species. The allelopathic agent present in black walnut is a phenolic compound called juglone (5-hydroxy-1,4-napthoquinone) (**Table 1**). Juglone, previously known as nucin was first isolated and identified in 1856 (Vogel and Reischauer, 1856), but Davis in 1928 proved the toxic effects of synthetic juglone on tomato and alfalfa plants. Since then, numerous studies have reported the allelopathic effects of juglone on various vegetables, field crops, fruit trees, ornamental species, and medicinal plants (Strugstad and Despotovski, 2012). In a greenhouse experiment, Ercisli et al. (2011) reported that 1 mM juglone inhibited the growth of strawberry plants and had a negative impact on plant nutrient uptake. In another study, hydroponically grown corn, and soybean (*Glycine max* [L.] Merr.) seedlings were examined at various concentrations of juglone (10^-4^, 10^-5^, 10^-6^ M). Exposure to juglone (>10 µM) has been shown to decrease the cell wall-bound peroxidase activities, root length, and dry mass in the affected plant (Bohm et al., 2006). Black walnut extract (NatureCur^®^, Redox Chemicals LLC, Burley, ID, USA) showed the potential to be a PRE and POST emergent bioherbicide against horseweed (*Conyza canadensis* (L.) Cronquist), hairy fleabane (*Conyza bonariensis* (L.) Cronquist), purslane (*Portulaca oleracea* L.), and tall annual morning glory (*Ipomoea purpurea* (L.) Roth) (Shrestha, 2009). This study was conducted in laboratory, greenhouse and in field to test the PRE and POST emergence ability of juglone.The herbicidal activity of juglone was evaluated on the growth of four weed species, wild mustard (*Sinapis arvensis* L.), creeping thistle (*Cirsium arvense* (L.) Scop.), field poppy (*Papaver rhoeas* L.), and henbit (*Lamium amplexicaule* L.). Juglone at a high concentration of 1.15 – 5.74 mM completely controlled the growth of field poppy and significantly reduced the elongation and fresh weight of all the remaining weed species (Topal et al., 2007). Juglone has a great potential to be used as a natural herbicide as it has been shown to provide excellent control of various weed species.

**Table 1:** Examples of allelopathic plants with their potent allelochemical against sensitive plants.

Allelopathic plant	Main allelochemical	Sensitive/target plants	Reference
Black walnut (*Juglans nigra* L.)	Juglone	Horseweed (*Conyza canadensis* (L.) Cronquist), hairy fleabane (*Conyza bonariensis* (L.) Cronquist), purslane (*Portulaca oleracea* L.), and tall annual morning glory (*Ipomoea purpurea* (L.) Roth)	Shrestha, 2009
Eucalyptus (*Eucalyptus* spp.)	1,8-cineol	Common amaranth (*Amaranthus retroflexus* L.) and common purslane (*Portulaca oleracea* L.)	Azizi and Fuji, 2006
Tree of heaven (*Ailanthus altissima* (Mill.) Swingle)	Ailanthone	Common amaranth (*Amaranthus retroflexus* L.), garden cress (Lepidium sativum L.), foxtail (*Setaria glauca* (L.) P.Beauv.), barnyard grass (*Echinochloa crus-galli*(L.) P.Beauv.), and corn (*Zea mays* L.)	Heisey, 1996
Fine fescue grasses (*Festuca* spp.)	M-tyrosine	Rape (*Brassica nigra* (L.) K.Koch), birdsfoot trefoil (*Lotus corniculatus* L.), large crabgrass (*Digitaria sanguinalis* (L.) Scop.), white clover (*Trifolium repens* L.), and common dandelion (*Taraxacum officinale* F.H. Wigg.)	Peters, 1968; Stephenson and Posler, 1988; Bertin et al., 2007
Rice (*Oryza sativa* L.)	Tricin and Momilactone B	Jungle rice (*Echinochloa colona* (L.) Link), monarch redstem (*Ammannia baccifera* L.), and gulf leaf flower (*Phyllanthus fraternus* G.L.Webster), flatsedge (*Cyperus iria L.*)	Khan and Vaishya, 1992; Lin et al., 1992
Sorghum (*Sorghum bicolor* (L.) Moench)	Sorgoleone	False cleavers (*Galium spurium* L.), Japanese dock (*Rumex japonicus* Houtt.), Indian jointvetch (*Aeschynomene indica* L.), and common amaranth (*Amaranthus retroflexus* L.)	Uddin M. et al., 2013
Ragweed parthenium *(Parthenium hysterophorus* L.)	Parthenin	Green amaranth (*Amaranthus viridis* L.), coffee senna (*Cassia occidentalis* L.), barnyard grass (*Echinochloa crus-galli* (L.) P.Beauv.), and small-seeded canary grass (*Phalaris minor* Retz.)	Batish et al., 2007
Common lantana (*Lantana camara* L.)	Lantadene A and lantadene B	Water hyacinths (*Pontederia crassipes* Mart.), *Microcystis aeruginosa*, small-seeded canary grass (Phalaris minor Retz.), common wild oat (*Avena fatua* L.), lamb’s quarters (*Chenopodium* *album* L.), toothed dock (*Rumex dentatus* L.)	Kong et al., 2006
morning glory (*Ipomoea tricolor* Cav.)	Tricolorin A	Annual Ryegrass (*Lolium mutliflorum* Lam. and common wheat (*Triticum vulgare* Vill.) tomatillo (*Physalis ixocarpa* Brot. ex Hornem.) and Egyptian clover (*Trifolium alexandrinum* L.)	Lotina-Hennsen et al., 2013
Annual wormwood (*Artemisia annua* L.)	Arteether and Artemisinin	Lettuce (*Lactuca sativa* L.), common amaranth (*Amaranthus retroflexus* L.), pitted morning-glory (*Ipomoea lacunose* L.), purslane (*Portulaca oleracea* L.).	Duke et al., 1987
Giant ragweed (*Ambrosia trifida* L.)	1α-angeloyloxycarotol	Wheat (*Triticum aestivum* L.), Sunflower (*Helianthus annuus* L.)	Kong et al., 2007; Šućur et al., 2021
Bottlebrush (*Callistemon citrinus* (Curtis) Skeels)	Leptospermone	Hairy crabgrass (*Digitaria sanguinalis* (L.) Scop.), yellow foxtail *(Setaria glauca* (L.) P. Beauv.), common amaranth (*Amaranthus retroflexus* L.), California red oat (*Avena sativa* L.), Indian mustard (*Brassica juncea* L.) and curly dock (*Rumex crispus* L.)	Cornes, 2005
Long pepper (*Piper longum* L.)	Sarmentine	Field bindweed (*Convolvulus arvensis* L.), wild mustard (*Sinapis arvensis* L.), horseweed (*Conyza canadensis* (L.) Cronquist) and sprangletop (*Leptochloa chinensis* (L.) Nees)	Dayan et al., 2015
Neem (*Azadirachta indica* A.Juss.)	Nimbolide B, Nimbic acid B	Garden cress (*Lepidium sativum* L.), lettuce (*Lactuca sativa* L.), alfalfa (*Medicago sativa* L.)	Salam and Kato-Noguchi, 2010
Sunflower *(Helianthus annuus* L.*)*	Heliannuols	Hairy crabgrass (*Digitaria sanguinalis* (L.) Scop.) prickly sida (*Sida spinosa* L.), and common tumbleweed (*Amaranthus album* L.)	Frans and Semidey, 1992
Crabgrass (*Digitaria sanguinalis* (L.) Scop.)	Veratric acid, Maltol, and (−)-loliolide	Wheat (T*riticum aestivum* L.), maize (*Zea mays* L.), and soybean (*Glycine max* (L.) Merr.)	Zhou et al., 2013

### Eucalyptus (*Eucalyptus* spp.)

**Figure 2:**
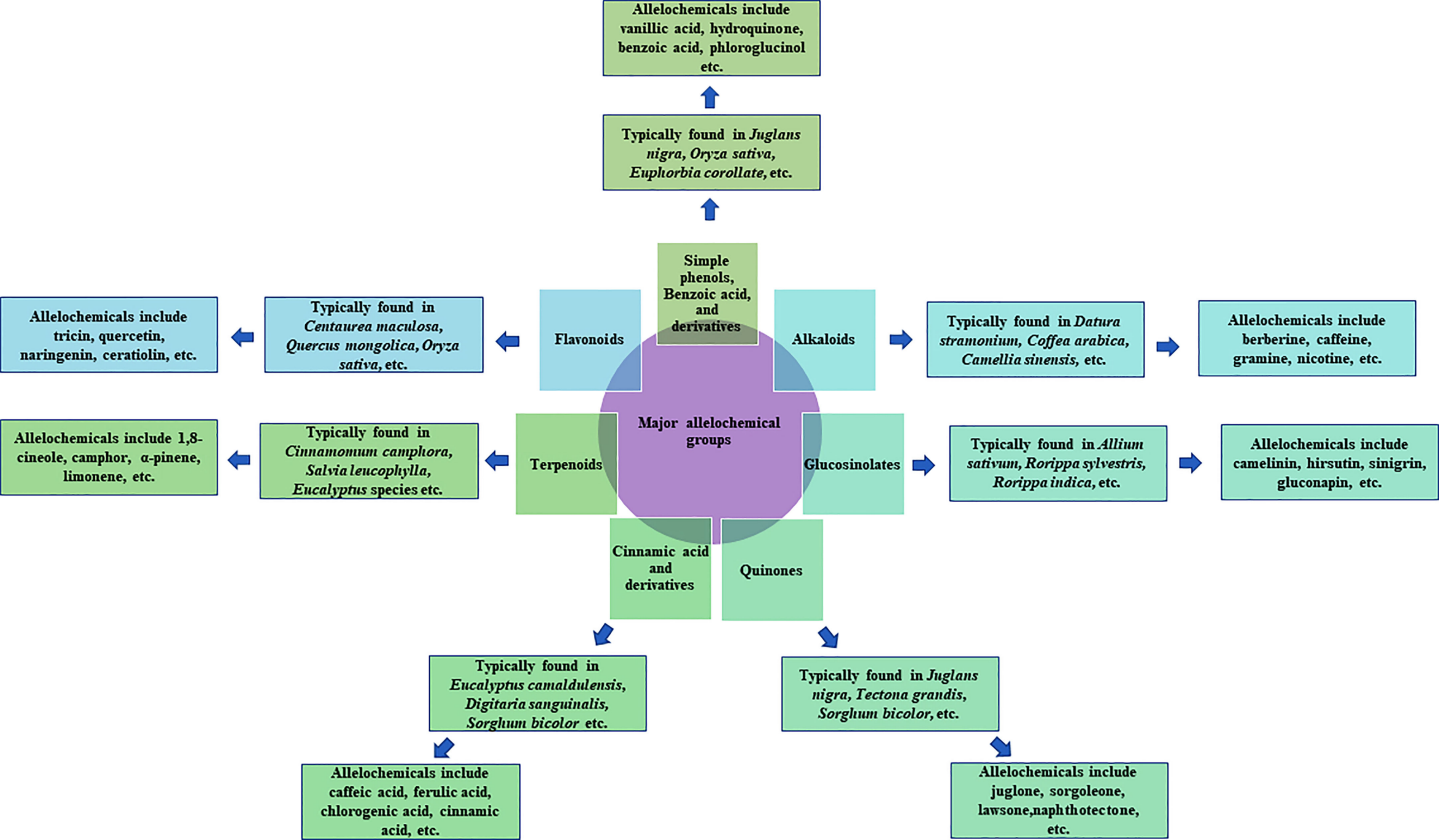
Selected seven allelochemicals from the 14 categories, their sources, and the allelochemicals present in them.

The allelopathic effects of the eucalyptus species have been investigated extensively (Sasikumar et al., 2002; Bajwa and Nazi, 2005; El-Khawas and Shehata, 2005). Phenolic acids and volatile terpenes are the allelochemicals present in the leaves, bark, and roots of *Eucalyptus* spp. (May and Ash, 1990). The foliage of the species also contains a variety of oils and resins that may have a direct or indirect effect on the neighboring plants, seeds, or microbes (Ruwanza et al., 2014). Volatile terpenes that act as allelochemicals, such as 1,8-cineol, limonene, α- and β-pinene have been reported present in the species (Muller et al., 1964) (**Figure 2**). In a greenhouse study, Babu and Kandasamy (1997) noted that leachates from fresh leaves of bluegum eucalyptus (*Eucalyptus globulus* Labill.) at a concentration of 20% (w/v) and 40% (w/v) reduced the resprouting of purple nutsedge (*Cyperus rotundus* L.) by 57%-68% and bermudagrass (*Cynodon dactylon* (L.) Pers.) by 82%-89%. In another study, the essential oils from bluegum eucalyptus reduced the growth of bermudagrass by 66% at a concentration of 25% (v/v) (Daneshmandi and Azizi, 2009). Similar results were observed by Azizi and Fuji (2006) in a petri dish experiment, eucalyptus essential oils at a concentration of 0.2% (v/v) and 0.5% (v/v) reduced the germination of Common amaranth (*Amaranthus retroflexus* L.) and common purslane by 80% and 90% respectively. One of the major allelopathic compounds present in eucalyptus leaf is 1,8-cineol (**Table 1**). 1,8-cineol can decrease germination, reduce root growth, and inhibit mitosis (Baum et al., 1998; Romagni et al., 2000).

### Tree of heaven (*Ailanthus altissima* (Mill.) Swingle)

The toxic activity of the tree of heaven was first confirmed by Voigt and Mergen (1962) who observed that the extract from foliage and stem was toxic to the neighboring plants. The plant extracts and essential oils from different parts of the plant consist of alkaloids, terpenoids, steroids, flavonoids, phenolic derivatives, and quassinoids (Albouchi et al., 2013; Kim et al., 2015; Ni et al., 2018). A study comparing the phytotoxicity of different parts of the plant showed that toxic activity was highest in the root bark and lowest in wood (Heisey, 1990a). In a container study, the oven-dried root bark of the tree of heaven mixed in the soil was shown to decrease the emergence, biomass, and survival of garden cress (*Lepidium sativum* L.) (Heisey, 1990b). A major allelochemical present in the tree of heaven is a quassinoid compound called ailanthone (Heisey, 1996; Sladonja et al., 2015) (**Table 1**). Ailanthone has shown herbicidal activity both pre- and postemergence by inhibiting the germination and growth of monocots and dicots. A greenhouse trial with purified ailanthone showed strong PRE and POST emergence control at application rates down to 0.5 kg/ha. The postemergence activity was strong, even at a low rate of 0.5 kg/ha, inhibiting growth or killing young seedlings of redroot pigweed (*Amaranthus retroflexus* L.), garden cress, foxtail (*Setaria glauca* (L.) P.Beauv), barnyard grass (*Echinochloa crusgalli* (L.) P.Beauv.), and corn (*Zea mays* L.) (Heisey, 1996). It was interesting to note that the tree of heaven seedling did not show any damage from the post-emergence application of ailanthone, indicating a presence of a protective mechanism to prevent autotoxicity (Heisey, 1996). Ailantone is not commercially used as an herbicide (Bhowmik and Inderjit, 2003), since its separation and purification costs are high, it degrades rapidly in the soil and it is nonselective (Heisey, 1996; Heisey, 1999). But with further research, it should have great potential to be used as a natural herbicide, especially for organic farmers and home gardeners.

### Fine fescue grasses (*Festuca* spp.)

Fine fescue grasses are known to displace the neighboring plants by releasing allelochemicals through roots into the soil. Several studies have focused on the weed management potential of fine fescue grasses through their toxic root leachates (Bertin et al., 2009; Peters and Mohammed Zam, 1981). Aqueous extracts from dried tall fescue (*Festuca arundinacea* Schreb.) shoots and roots have been shown to reduce the growth of rape (*Brassica nigra* (L.) K.Koch) and decrease germination, growth, and yield of birdsfoot trefoil (*Lotus corniculatus* L.) in both greenhouse and field experiments (Peters, 1968; Stephenson and Posler, 1988). Weston (1990) showed that creeping red fescue (*Festuca rubra* L.) had strong weed suppressive ability when used as a living mulch or as killed sod in a no-tillage field experiment. The major allelochemical released through the root exudates of fine fescue grass is a non-protein amino acid called m-tyrosine (Bertin et al., 2007). M-tyrosine has been demonstrated to inhibit the germination and growth of large crabgrass (*Digitaria sanguinalis* (L.) Scop.), white clover (*Trifolium repens* L.), and common dandelion (*Taraxacum officinale* F.H. Wigg.) (Bertin et al., 2007). M-tyrosine acts as a natural herbicide that affects the post-germination development and early establishment of the neighboring plants. It has been shown to impact cell division and cell elongation in several higher plant species (Bertin et al., 2007). With more support and research, M-tyrosine has the potential to be developed as a pre-emergent soil-applied natural herbicide.

### Rice (Oryza sativa L.)

The allelopathic potential of rice has received a great deal of attention. Several cultivars of rice have the allelopathic potential that has been well documented both in the field and laboratory studies (Olofsdotter et al., 1999). In a field experiment performed with 5,000 rice accessions for allelopathic activity on duck salad *(Heteranthera limosa* (Sw.) Willd.), researchers found approximately 191 accessions with evident allelopathic activity (Dilday et al., 1991). Another study reported that 45 out of 1,000 screened rice cultivars showed allelopathic activity against barnyard grass or monochorea (*Monochoria vaginalis* (Burm.f.) C.Presl) (Olofsdotter et al., 1997). Lee et al., 2004 evaluated the allelopathic activities of 749 rice cultivars and reported that japonica rice cultivar possessed stronger allelopathic potential against root growth of barnyard grass. Many phytotoxic compounds from several chemical classes, such as fatty acids, benzoxazinoids, indoles, phenolic acids, phenylalkanoic acids, and terpenoids have been found in rice extracts (Belz, 2007). The key allelochemicals found in allelopathic rice cultivars are tricin and momilactone B (Kato-Noguchi and Ino, 2003). Several phenolic acids, such as p-hydroxybenzoic, vanillic, ferulic, o-hydroxy phenylacetic, and syringic acid were found in rice residues under waterlogged conditions (Chou et al., 1981). Rice residues incorporated in soil at a depth of 5 to 6 cm at 5 ton/ha reduced the growth of jungle rice (*Echinochloa colona* (L.) Link), monarch redstem (*Ammannia baccifera* L.), and gulf leaf flower (*Phyllanthus fraternus* G.L.Webster) (Khan and Vaishya, 1992). Rice residues from high allelopathic cultivars decreased the growth of flatsedge (*Cyperus iria* L.) similar to the application of propanil and bentazon herbicides (Lin et al., 1992). Rice residues contain phenolic acids, such as p-hydroxybenzoic, p-coumaric, syringic vanillic, ferulic, and o-hydroxyphenylacetic acids and other autotoxins (Chou and Lin, 1976). These compounds have been shown to inhibit paddy weed growth and increase rice yield (Xuan et al., 2005). The incorporation of rice straw, rice flour, and hull can be directly used as a component of an integrated weed management program. Further research on allelochemical extraction from allelopathic rice cultivars and evaluating their mode of action will open up the chances of utilizing rice allelopathy for weed management.

### Sorghum (*Sorghum bicolor* (L.) Moench)

The weed suppressive ability of sorghum is due to the presence of hydrophilic compounds, phenolic acids, and their aldehyde derivatives, as well as hydrophobic substances, such as sorgoleone (Czarnota et al., 2003). In 1986, Netzly and Butler isolated sorgoleone from hydrophobic root exudates of sorghum. About 90% of compounds present in the root exudates of sorghum comprises of sorgoleone (Czarnota et al., 2003). Sorgoleone is synthesized in the root hair cells of sorghum (Yang et al., 2004). Sorgoleone has been characterized as a potent bioherbicide as it can suppress many weed species. It has been shown to have greater activity than that of other allelochemicals such as juglone, and other phenolics and terpenoids (Uddin M. et al., 2013). Sorghum can be applied in various forms to control weeds, such as surface mulch, mixed in the soil, extract spray, or inter-cropping. The incorporation of sorghum roots, stems, and leaves in the soil has been shown to suppress weed biomass by 25-50% (Cheema, 1988). Foliar addition of sorghum water extract known as sorgaab reduced the density and dry weight of purple nutsedge by 44 and 67% respectively and increased maize grain yield by 44% (Cheema et al., 2004). A wettable powder formulation with a 4.6% active ingredient (sorgoleone) was prepared by Uddin M. et al. (2013). Sorgoleone at 0.2 g active ingredient completely suppressed the germination and growth of false cleavers (*Galium spurium* L.), Japanese dock (*Rumex japonicus* Houtt.), Indian jointvetch (*Aeschynomene indica* L.), and common amaranth. Post-emergence application of the wettable powder inhibited weed growth by 20-25% higher than the pre-emergence application. Nine other allelochemicals, namely benzoic, p-hydroxybenzoic, vanillic, m-coumaric, p-coumaric, gallic, caffeic, ferulic, and chlorogenic acids were also identified by Cheema, 1988. A multidisciplinary approach to incorporate sorghum crops, residues, or allelochemicals for strategic weed management can be an alternative with great potential.

### Wheat (Triticum aestivum L.)

Wheat, like rice and sorghum has been comprehensively studied for its allelopathic potential. There have been number of studies on the allelopathic potential of weed residues, straw, seedlings and aquaeous extract (Steinsiek et al., 1982; Wu et al., 2000). It was reported that the water extract from wheat grass was phytotoxic against ivyleaf morning glory (*Ipomoea hederacea* (L.) Jacq.), velvetleaf (*Abutilon theophrasti* Medic.), and pitted morningglory (*Ipomoea lacunosa* L.) (Steinsiek et al., 1982). Major allelochemical groups present in wheat include polyphenols and hydroxamic acids (Niemeyer, 1988; Krogh et al., 2006). Main phenolic acids present in wheat mulch and in its surrounding soil include *p*-hydroxybenzoic, vanillic, *p-*coumaric, syringic, and ferulic acids (Lodhi et al., 1987). Additionally, hydroxamic acids (benzoxazinoids) and lactams such as 2,4- dihydroxy-1,4-benzoxazin-3-one (DIBOA), 2,4-dihydroxy7-methoxy-1,4-benzoxazin-3-one (DIMBOA), 2-hydroxy-1,4-benzoxazin-3-one (HBOA), and 2-hydroxy-7-methoxy-1,4-benzoxazin-3-one (HMBOA) have been identified as main allelochemicals in wheat. DIBOA was further metabolized to a more active compound called benzoxazolin-2-one (BOA). BOA has shown to cause inhibition of germination and reduction of seedling growth in many plant species. Burgos and Talbert (2000) reported that DIBOA was seven times more toxic to root growth of weed species than BOA. Wheat shows allelopathic potential due to the presence of various allelochemicals, but more research needs to be focused on understanding the genetic control of wheat allelopathy. Further, the selection and breeding of wheat cultivars with higher allelopathic effect against weed species need more attention.

### Miscellaneous weed species

Many weed species consist of special allelochemicals that can be used to suppress the germination and growth of other weed species. Ragweed parthenium *(Parthenium hysterophorus* L.) contains a sesquiterpene lactone of pseudoguanolide nature called parthenin in various parts of the plant (Kanchan and Jayachandra, 1980), with the greatest concentration present in the leaves (Kanchan, 1975). Pre- and post-emergent application of parthenin reduced the seedling growth and dry weight of green amaranth (*Amaranthus viridis* L.), coffee senna (*Cassia occidentalis* L.), barnyard grass, and small-seeded canary grass (*Phalaris minor* Retz.) (Batish et al., 2007). Other phenolic compounds such as caffeic, vanillic, ferulic, chlorogenic, and anisic acid are also isolated from ragweed parthenium (Kanchan, 1975; Kanchan and Jayachandra, 1980). Common lantana (*Lantana camara* L.) is an obnoxious weed with allelochemicals present in leaves, stems, roots, fruits, and flowers (Wahab, 2004). The allelochemicals present in common lantana include mono and sesquiterpenes, flavonoids, iridoid glycoside, furanonaphoquinones, sthsteroids triterpenes, and diterpenes (Sharma et al., 2007). Lantadene A and lantadene B are more potent allelochemicals present in common lantana (Sharma et al., 2007). In tropical areas of Mexico, morning glory (*Ipomoea tricolor* Cav.) is used as green manure to manage weeds (Anaya et al., 1990). Tricolorin A, a resin glycoside is the major allelopathic compound present in morning glory (Pereda-Miranda et al., 1993). Several species of *Artemisia* spp. have shown allelopathic activity in different field settings. The major compounds present in common mugwort (*Artemisia vulgaris* L.) are alpha thujone, a monoterpene, as well as 20 minor components, including numerous sesquiterpenes (Dung et al., 1992). Annual wormwood (*Artemisia annua* L.) contains sesquiterpene lactones called arteether and artemisinin, which have shown to be a potent inhibitor of seed germination and plant growth (Duke et al., 1987). Giant ragweed (*Ambrosia trifida* L.) contains a carotene type sesquiterpene called 1α-angeloyloxycarotol that acts as a strong allelochemical (Kong et al., 2007) (**Table 2**). Brazilian pepper (*Schinus terebinthifolia* Raddi) an exotic invasive species contains sequiterpenes that have been shown to inhibit radicle growth of lettuce (*Lactuca sativa* L.) and cucumber (*Cucumis sativus* L.) (Barbosa et al., 2007). Many other weed species contain special types of allelochemicals. These allelochemicals may possess novel modes of action and can be utilized as herbicide templates, to discover future herbicides.

**Table 2:** Example of allelopathic crop and main crop intercropping system.

Allelopathic crop	Main crop	Weed species	References
Cowpea (*Vigna unguiculata (L.) Walp.*)	Maize (*Zea mays* L.)	Barnyardgrass (*Echinochloa colona* (L.) Link), purslane (*Portulaca oleracea* L.), tossa jute (*Chorchorus olitorius L.)*, crowfoot grass *(Dactyloctenium aegyptium* (L.) Willd*)*	Saudy, 2015
Barley (*Hordeum vulgare* L.)	Pea (*Pisum sativum* L.)	Common lambsquater (*Chenopodium album* L.), wild mustard (*Sinapis arvensis* L.)	Corre-Hellou et al., 2011
Sorghum (*Sorghum bicolor* (L.) Moench), soybean (*Glycine max* (L.) Merr.) and sesame (*Sesamum indicum* L.)	Cotton (*Gossypium hirsutum* L.)	Purple nutsedge (*Cyperus rotundus* L.)	Iqbal et al., 2007
Sorghum (*Sorghum bicolor* (L.) Moench)	Maize (*Zea mays* L.)	Purple nutsedge (*Cyperus rotundus* L.), field bindweed (*Fallopia convolvulus* L.) and horse purslane (*Trianthema portulacastrum* L.)	Khalil et al., 2010
False flax (*Camelina sativa* L.)	Pea (*Pisum sativum* L.)	Field bindweed (*Fallopia convolvulus* L.), sow thistle (*Sonchus oleraceus* L.), chamomile (*Matricaria recutita* L.)	Saucke and Ackermann, 2006
Sorghum (*Sorghum bicolor* (L.) Moench)	Cotton (*Gossypium hirsutum* L.)	Purple nutsedge (*Cyperus rotundus* L.)	Iqbal et al., 2007
Chickpea (*Cicer arietinum* L.)	Wheat (*Triticum aestivum* L.)	Common lambsquater (*Chenopodium album* L.), burr medic (*Medicago polymorpha* L.), sweet clover (*Melilotus indicus* (L.) All.), scarlet pimpernel (*Anagallis arvensis* L.), swine watercress (*Lepidium didymum* L.)	Banik et al., 2006
Maize (*Zea mays* L.)	Cassava (*Manihot esculenta* Crantz)	Redroot pigweeed (*Amaranthus retroflexus* L.), giant foxtail (*Setaria* *faberi* Herrm.), bermudagrass (*Cynodon dactylon* (L.) Pers.)	Olasantan et al., 1994
Spanish tick-clover (*Desmodium uncinatum* [Jacq.] DC.), green leaf desmodium (*Desmodium intortum* [Mill.] Urb.)	Maize (*Zea mays* L.)	Giant witchweed (*Striga hermonthica* [Del.])	Khan et al., 2002
Canola *(Bassica napus* L.)	Wheat (*Triticum aestivum* L.)	littleseed canarygrass (*Phalaris minor* Retz.), broad-leaved duck (*Rumex obtusifolius* L.), swine watercress (*Lepidium didymum* L.), and common lambsquarters (*Chenopodium album* L.)	Naeem, 2011

## Application of allelopathy in agriculture

### Intercropping

Intercropping is the practice of growing different crops together at the same time in the same field. Intercropping with allelopathic species has great potential to be suppress weed in an environmentally friendly approach. Intercropping involves growing compatible crops together to improve yield, diversify the farm and provides economic benefits. Furthermore, it is also a great way for improving land, water, nutrient and light efficiency. Factors, such as weed-crop competition, release of allelochemical and effect of shade can be used by allelopathic intercrops to control weeds (Baumann et al., 2002). Intercropping cowpea with maize has shown to reduce the growth of jungle rice, purslane., jute mallow (*Chorchorus olitorius* L.), and Egyptian crowfoot grass (*Dactyloctenium aegyptium* (L.) Willd.]) (Saudy, 2015) (**Table 2**). Intercropping sesame (*Sesamum indicum* L.), soybean and sorghum in cotton (*Gossypium hirsutum* L.) decreased the purple nutsedge density by 70%-96% and dry biomass by 71%-97% (Iqbal et al., 2007). Bulson et al. (1997) reported that intercropping field beans (*Vicia faba* L.) and wheat can reduce growth of weeds and improve yield compared to sole cropping of wheat. Intercropping maize with Spanish tick-clover (*Desmodium uncinatum* [Jacq.] DC.) and green leaf desmodium (*Desmodium intortum* [Mill.] Urb.) decreased the growth and density of giant witchweed (*Striga hermonthica* [Del.]) than the sole maize crop (Khan et al., 2002). Intercropping white clover (*Trifolium repens* L.), black medic (*Medicago lupulina* L.), alfalfa, and red clover (*Trifolium pratense* L.) in wheat crop was much effective in controlling various weed species and improve wheat yield (Naeem, 2011). Rad et al., 2020 evaluated intercropping sorghum with different ratios of hairy vetch (*Vicia villosa* Roth) and lathyrus (*Lathyrus sativus* L.) with three different strategies of no weed control, full weed control and hand weeding. The results showed that the highest sorghum yield was obtained with soghum and 33% hairy vetch while lowest in sorghum and 100% lathyrus. Sorghum with 100% lathyrus showed the highest weeding efficiency. In another study, intercropping wheat with white clover along with high N availability resulted in decrease in weed shoot dry matter, increase in cover crop biomass and improved N accumulation with high wheat yield and protein content (Vrignon-Brenas et al., 2018). Intercroping wheat with canola (*Bassica napus* L.) was successful in decreasing density and biomass of littleseed canarygrass, broad-leaved duck (*Rumex obtusifolius* L.), swine cress swine watercress (*Lepidium didymum* L.), and common lambsquarters (*Chenopodium album* L.) (Naeem, 2011). In general, in an intercropping system, the available resources, such as water, nutrient, and light are efficiently utilized by two or more crops and thus weeds do not get available resources for their growth (Liebman and Dyck, 1993). The success of weed suppression ability from intercropping through allelopathic plants can be variable. But given the potential benefits of intercropping with allelopathic plants, further examination of allelopathic plants in combination of main crops is required.

### Crop rotation

Crop rotation is a method of growing different crops in a systematic and sequential way in the same field over a growing season. Crop rotation has many benefits ranging from maintaining soil structure, adding organic matter, reducing soil erosion associated with monoculture system. But one of main benefits of crop rotation is suppressing weeds, disease pathogens and insect pests (Peters et al., 2003; Farooq et al., 2011). In crop rotation, allelopathic crops release allelochemicals through roots or *via* decomposition of crop residue to suppress weeds and other pests. Other factors, such as unique root systems, distinct time of sowing and harvesting, varying cultural techniques for crop management may also be responsible for weed suppression (Peters et al., 2003). Different sowing and harvesting dates among rotating cool and warm season crops can prevent weed establishment or seed production. Recent research has shown that allelopathic crops add allelochemicals in the soil that results in weed suppression for the subsequent crop. Dmitrović et al. (2015) reported that the growth of barnyardgrass in soil after the harvest of an allelopathic rice cultivar PI312777 was suppressed compared to the soil of a non-allelopathic rice cultivar. Many weed species are specific to a particular crop. For example, wild oat is commonly found in wheat field, cocklebur and velvetleaf in soybean, barnyardgrass, foxtail and fall panicum in corn or sorghum field (Unger and McCalla, 1980). With planning crop rotation, crops that can effectively compete against certain weeds are included in rotation. A field with troublesome winter weed species are rotated with spring or summer crops, whereas a field with summer annual weed issues are rotated with winter grain crops. A major cropping system in many Asian countries is rice-wheat system. This system is heavily infested with weeds and largely controlled *via* herbicides. Use of allelopathic crops such as pearl millet (*Pennisetum glaucum* L.), maize and sorghum after wheat harvest and before rice transplantation has shown to be an effective weed control method (Peters et al., 2003). Integration of allelopathic crops as smothering crops that can grow fast with thick canopy can provide additional weed control. Crops such as sudan grass (*Sorghum sudanense* L.), common buckwheat (*Fagopyrum esculentim* Moench), rye, barley, sunflower (*Helianthus annuus* L.), sweet clover, cowpea can effectively smother various weed species (Cheema et al., 2012).

Although incorporation of allelopathic crops in crop rotation is an effective weed control method, there has been instances where negative consequences with allelopathic crop has been observed. Wheat growth was delayed by sorghum root exudates (Ben-Hammouda et al., 1995). Similarly, maize development was delayed and reduced in a rye-maize double cropping system (Raimbault et al., 1990). Further investigation of crop rotation with allelopathic crops and screening efficient combinations for weed control is currently required.

### Cover crops

The integration of cover crops is rising in many agriculture practices as it reduces the use of herbicide and acts as an alternative to tillage for controlling weeds. The main goal of using cover crops is to replace the weed species growing in between crop rows with useful and manageable cover crops (Samarajeewa et al., 2006). Cover crops are also known as living mulch, green manure, smother crop or catch crop (Mennan et al., 2020). Cover crops are either grown during fallow period or along with cash crop during the growing season. Cover crops are not grown for profits but rather they are grown for various of their ecological benefits. The ecological benefits include weed suppression, soil conservation, improvement in soil fertility, attract beneficial insects, maintain soil moisture, decrease soil erosion, and improve soil structure (Mennan et al., 2020). A major concern with cover crop is yield reduction, but with right planning and proper management at cover crops can not only suppress weeds but improve crop yield (Oliveira et al., 2019). For example, growing alfalfa in wheat production field as a green mulch increased soil N contents due to the fixation of atmospheric N_2_, resulting in high grain yield of wheat and also suppressed weeds (Ominski et al., 1999; Wiens et al., 2006).

One of the major benefits of cover crops is its ability to reduce weed growth. Cover crops initially act as a living mulch, blocking empty space for weeds to grow and after their termination as a plant residue (Osipitan et al., 2019). Cover crops provide weed suppression through allelopathy, competition for resources and act as a physical barrier. Some of the important cover crops include sunhemp (*Crotalaria juncea* L.), yellow sweet clover (*Melilotus officinalis* (L.) Pall.), sorghum, cowpea, alfalfa, ryegrass, red clover, white mustard (*Sinapis alba* L.), wheat and cereal rye (*Secale cereale* L.) (Farooq et al., 2011; Tursun et al., 2018). Growing cover crop of rye, barley, wheat or sorghum to a height of 40-50 cm, followed by their desiccation and allowing the residues on the soil surface as crop residue has resulted in up to 95% control of several weed species for 30–60-day period (Putnam et al., 1983). Einhellig and Leather (1988) suggested use of sorghum as a cover crop for suppression of broadleaf weeds. Similarly, velvetbean (*Mucuna prusens* (L.) DC.) was reported as an effective cover crop to smother weeds (Fujii et al., 1992). L-DOPA (L-3,4-dihydroxyphenylalanine) was identified as the allelochemical present in velvetbean responsible for weed suppression. Barley grown as cover crop in soybean field has shown to decrease weed growth of crabgrass and barnyardgrass (Kobayashi et al., 2004). There are many allelopathic cover crops that have been found to be effective in suppressing weeds. Mixtures of these allelopathic crops as cover crops can be more effective in controlling weeds compared to use of a single cover crop. Use of a mixture of allelopathic cover crops can result in release of diverse allelochemicals and higher biomass that can synergistically suppress weeds efficiently.

### Mulching

Mulching is a process of adding any material to the surface of soil to reduce weed growth, improve soil moisture, and reduce surface runoff (Adekalu et al., 2007; Chalker-Scott, 2007). Mulching reduces weed germination and growth by light exclusion (Richardson et al., 2008), acting as a physical barrier (Marble, 2015), and reducing available moisture in the top layer (Jordán et al., 2010). More importantly, many mulch materials control weeds by leaching allelochemicals (Chalker-Scott, 2007). Use of mulch material from allelopathic crop residues also improves agriculture sustainability by adding organic matter to soil, maintaining soil temperature, controlling topsoil erosion, conserving soil moisture, and supporting the microbes in the soil (Tiquia et al., 2002).

Some studies have investigated the allelopathic properties of common nursery mulches. Six common landscape mulch materials made of cypress, eucalyptus, pine bark, pine straw, *Melaleuca*, and a utility trimming mulch (mix of multiple species) were evaluated for their chemical, allelopathic and decomposition properties (Duryea et al., 1999). Results showed that all the mulch materials contained hydroxylated aromatic compounds, with the highest amount present in the utility trimming mulch and the lowest in *Melaleuca*, pine bark, and pine straw. Results also showed that lettuce seed germination was inhibited by the water-soluble extracts from pine bark and utility trimming mulch. Shredded or chopped leaves of eucalyptus species are known to be toxic to seedlings (Kohli and Singh, 1991). In another study, exudates from wood chips of southern redcedar (*Juniperus silicicola* (Small) L.H.Bailey), red maple (*Acer rubrum* L.), swamp chestnut oak (*Quercus michauxii* Nutt.), neem (*Azadirachta indica* A.Juss.), and magnolia (*Magnolia grandiflora* L.) inhibited radicle growth in germinating lettuce (Rathinasabapathi et al., 2005). The leachates from decomposing eucalyptus litter were shown to reduce the germination and radicle growth of lettuce (Molina et al., 1991). Downer and Faber (2005) showed that mulch prepared from fresh and composted sugar gum (*Eucalyptus cladocalyx* F.Muell.) decreased coverage and abundance of annual weeds.

In field studies, many crop residues used as mulch have been shown to suppress weeds *via* allelopathy (White et al., 1989; Batish et al., 2001). Crop residues made from wheat (*Triticum aestivum* L.), rice, sorghum, alfalafa (*Medicago sativa* L.), sunflower, and corn have shown evidence to suppress weed growth through allelopathy (Singh et al., 2003). Sorghum mulch has been shown to decrease purple nutsedge growth by 38-41% (Cheema et al., 2004). In a container study, incorporation of sorghum, sunflower, and brassica residues into the soil showed to suppress sprouting and seedling growth of purple nutsedge and horse purslane (Matloob et al., 2010). Mahmood and Cheema, 2004 reported that the density of purple nutsedge was decreased by 40%-45% when sorghum mulch was surface applied, and soil incorporated. There are several allelopathic mulch that have shown great weed control. Combination of more than one allelopathic mulch has also shown to be effective in weed control. Crop residues of brassica, sunflower, and sorghum are able to control the growth of horse purslane and purple nutsedge much better than sole application of these crop residues (Khaliq et al., 2011). A diversity of allelopathic mulch materials can also improve the ability to suppress weed growth due to presence of variety of allelochemicals. Research has shown that allelopathic plant residues used as mulch has value in controlling weed, improve crop yield and enhance soil quality.

### Allelopathic activity of water extract

Many of the secondary metabolites with allelopathic potential are water-soluble, and water acts as the carrier and medium for allelopathic activity (Farooq et al., 2011). Water-soluble allelochemical extract from different parts of allelopathic species, such as leaves, stems, roots, and seeds have great potential to be used for controlling weeds. It has been reported that there are about 400,000 compounds in plants with allelopathic potential, and only about 3% of these compounds have been identified for their herbicidal activity (Einhellig and Leather, 1988) (**Table 1**). These allelochemicals can control the germination and growth of weeds through various modes of action. For example, sorghum extract releases hydrophilic compounds, phenolic acids, and their aldehyde derivatives, as well as hydrophobic substances, such as sorgoleone (Czarnota et al., 2003). The water-soluble compounds in sorghum are phytotoxic to several weed species, such as small seed cannarygrass, lambsquaters, toothed dock (*Rumex dentatus* L.), and bindweed (*Convolvulus arvensis* L.) (Cheema, 1988). Similarly, different parts of the tree of heaven plant contain alkaloids, terpenoids, steroids, flavonoids, phenolic derivatives, and quassinoids (Albouchi et al., 2013; Kim et al., 2015; Ni et al., 2018). The water extract from fresh leaves of the tree of heaven showed to inhibit alfalfa germination and growth (Tsao et al., 2002). The aqueous extract from leaves of eucalyptus inhibited seed germination and decreased root and shoot, fresh and dry weight of maize (Khan et al., 2004). While a single plant water extract may be effective, combining these plant allelopathic water extracts may increase their efficacy. Application of water extract from sorghum, sunflower, and eucalyptus resulted in 70% weed control compared to the sole application of sorghum water extract (Cheema et al., 2003). The combined application of sorghum and sunflower water extract reduced the growth of horse purslane (*Trianthema portulacastrum* L.) by 66% (Azhar et al., 2010). Several studies have also shown that the combination of allelopathic water extract applied with herbicide can reduce the herbicide dosage substantially. The mixture of water extracts from sorghum, sunflower, and rice with a lower rate (1/2 of label rate) of pre-emergence herbicides decreased weed growth by 60-70% and reduced the herbicide dosage by 20-67% (Rehman et al., 2010). For weed control in cotton and maize, a half dose of atrazine in combination with sorghum water extract, controlled weeds similar to a full dose of atrazine (Iqbal et al., 2009). Iqbal and Cheema (2008) reported that pre-emergence application of sorghum water extract in combination with half and one-third dose of S-metolachlor was more successful in controlling purple nutsedge than the standard dose. The use of plant allelochemical water extract solely or in combination with other herbicides can add a new tool for weed management in agriculture. This would additionally offer environmental benefits as it would minimize the use of herbicides and other resistance issues. Different plant species have different types of allelochemicals, and this results in numerous allelopathic activities that can be explored. Allelopathic water extract from many of these species could be a promising means for weed control.

### Allelochemicals as natural herbicides

The need for environmentally safer herbicides and the difficulty of discovering a new mode of action coupled with an increase in herbicide-resistant weed strains have prompted the development of natural herbicides. Allelochemicals as natural herbicides can be of particular value for weed management since they offer new modes of action, more specific interactions with weeds, and are environmentally friendly. In addition, allelochemicals have been used as leads for the discovery of synthetic herbicides and can offer insights into new modes of action. Mesotrione was derived from a natural compound called leptospermone from the roots of bottle brush plant (*Callistemon citrinus* (Curtis) Skeels) (Mitchell et al., 2001). The triketone herbicides are derivatives of phytotoxin leptospermone. Leptospermone is also a major component present in the essential oil of the tea tree (*Leptospermum scoparium* J.R.Forst. & G.Forst.) (van Klink et al., 1999). Sulcotrione and mesotrione are post-emergent broadleaf herbicides that are based on the leptospermone structure template. Another example is cinmethylin, the first-ever commercial allelopathic herbicide that was derived from monoterpene 1,8-cineole. Monoterpene 1,8-cineole is present in essential oils of several plant species (Einhellig and Leather, 1988). Many other herbicides, such as AAL toxin, artemisinin, biolaphes, glufosinate, and dicamba have been developed from plant allelochemicals (Motmainna et al., 2021). Allelochemicals may not only provide us clues to new herbicide chemistry but also the natural compounds themselves that can be modified into active, selective, and persistent products.

It was also reported that there were 13 natural herbicides registered globally, nine of which were derived from fungi, three from bacteria, and only one from plant extract (Cordeau et al., 2016). Since then, six commercial natural herbicides derived from essential oils and/or their compounds were registered and available in the USA by 2020 (Verdeguer et al., 2020). The six commercial herbicides derived from essential oil are GreenMatch (55% d-limonene), Matratec (50% clove oil), WeedZap (45% clove oil + 45% cinnamon oil), GreenMatch EX (50% lemongrass oil), AvengerWeed Killer (70% d-limonene), and Weed Slayer (6% eugenol) (Verdeguer et al., 2020) (**Table 3**). One of the issues with natural herbicides is the need to apply large quantities to get the same result as compared to synthetic herbicides. This high quantities of allelochemicals can have adverse effect on the soil fauna, microbes, and the environment. These high quantities can not only negatively affect the environment, but it also makes the treatment expensive, even in high value production system. Additionally, many of the natural herbicides described in **Table 2** are non-selective herbicides and have very little crop selectivity. With the current concern over synthetic herbicides and public demand for organic produces, there is a need for more natural herbicides with better selectively in the market.

**Table 3:** Examples of commercial herbicides with allelochemicals as active ingredients. .

Commercial products	Company	Active ingredients	Acting	Reference
Avenger^®^ Weed Killer	Avenger Products, LLC, USA.	Citrus oil	Non-selective, postemergence herbicide	Verdeguer et al., 2020
Organic Weed & Grass killer™	Eco SMART Technologies, USA.	Citrus oil	Non-selective, postemergence herbicide	Dayan et al., 2009
WeedZap^®^	JH Biotech, Inc., USA.	Clove/Cinnamon oil	Non-selective, postemergence herbicide	Verdeguer et al., 2020
Beloukha^®^	Belchim Crop Protection USA.	Nonanoic acid and pelargonic acid	Non-selective leaf herbicide	Nguyen et al., 2016
Katoun^®^	Belchim Crop Protection USA.	Pelargonic acid	Non-selective contact herbicide	Korres et al., 2019
Scythe^®^ herbicide	DOW AgroSciences, USA.	Pelargonic acid	Non-selective contact herbicide	Dayan et al., 2009
AgraLawn CrabGrass Killer^®^	Avant-Grade Organics, USA.	Cinnamon bark	Selective postemergence crabgrass herbicide	Dayan and Duke, 2010
Concern Weed Prevention Plus^®^	Woodstream Corporation, USA.	Corn gluten (small peptides)	Preemergence control of broadleaf weeds	Dayan and Duke, 2010
GreenMatch™ EX	Pro Farm Group, USA.	Lemon grass oil	Non-selective, broad-spectrum herbicide.	Dayan et al., 2009
Weed Blitz^®^	Sustainable Formulations Group Pvt Ltd, Australia.	Pine oil	Preemergent weed control	Mani-López et al., 2017
Nature’s Way Organic Weed Spray ^®^	Yates Pty Ltd, Australia.	Acetic acid and clove oil	Non-selective, postemergence herbicide	Duke et al., 2018
Eco-Exempt™ and Eco-Smart ™	Eco SMART Technologies, USA.	2-phenethyl propionate and clove oil	Non-selective, postemergence herbicide	Duke et al., 2018
Weed Slayer Organic Herbicide	ANDAMAN AG Agro Research International LLC, USA	Eugenol (Clove Oil)	Non-selective, broad-spectrum herbicide.	Islam et al., 2018
Matran II ™	Eco SMART Technologies, USA.	Clove oil, wintergreen oil, butyl lactate, lecithin	Non-selective, postemergence herbicide	Islam et al., 2018
NatureCur^®^	Redox Chemicals, USA.	Black walnut extract	Selective pre- or post-emergence herbicide	Shrestha, 2009

Allelochemicals from allelopathic plants can be used to develop new natural herbicides or their chemistry can be used to learn about new target sites for herbicides. Several allelochemicals have been identified with the potential of being natural herbicides. Artemisinin is a sequiterpenoid lactone, a principle allelochemical present in annual wormwood. Artemisinin is a selective phytotoxin, and it has shown increased oxygen uptake and decreased chlorophyll content in the treated plants (Dayan et al., 1999). Ailanthone is a quassinoid lactone, a major allelochemical present in the tree of heaven (Heisey, 1996; Sladonja et al., 2015). It has the potential to be used as a post-emergence herbicide, but it degrades rapidly in the field, losing its effect after several days (Heisey, 1999). Sorgoleone, an allelochemical secreted from sorghum is a great example of a natural herbicide (Tibugari et al., 2020). Sorgoleone affects photosynthesis by disturbing the minerals and water uptake, especially in lower plants (Ashraf et al., 2017). The efficacy of sorgoleone as an herbicide has been compared to synthetic herbicides for commercial use (Jesudas et al., 2014). Sorgoleone has been shown to directly influence plant growth in laboratory, greenhouse, and field studies (Uddin M. R. et al., 2013). Juglone is another allelochemical that is a strong candidate for developing a natural herbicide. Juglone is a quinoid compound that is released from trees in the walnut family (Juglandaceae) including black walnut and English walnut (*Juglans regia* L.) (Rice, 1984). NatureCur^®^ (Redox Chemicals LLC, Burley, ID, USA) a botanical extract of the leaves, fruits, and branches of black walnut is available for commercial use (Shrestha, 2009) (**Table 3**). In recent years, tricin has received a great deal of attention for an allelochemical-based herbicide discovery (Kong et al., 2010). An isomer of tricin has been synthesized called aurone, which has shown much stronger herbicidal activity than tricin itself, guiding research towards a useful molecule for new herbicide discoveries. A series of aurone-derived compounds, including substituted aurones and benzothiazine derivatives, have been synthesized and several of these derivatives have shown great pre-emergent activity against weeds (Kong et al., 2010). Multicolored morning glory contains resin glycosides with tricolorin A as the main allelochemical (Pereda-Miranda et al., 1993). Tricolorin A has shown to be highly phytotoxic and is a potent inhibitor of plasma membrane adenosine triphosphate (Calera et al., 1995). The herbicidal effect of various essential oils has been widely studied as an alternative to synthetic herbicides. Many other types of allelochemicals ranging from phenolics, terpenoids, alkaloids, coumarins, tannins, flavonoids, steroids, and quinines are phytotoxic to other plant species (Kohli et al., 1997). In recent years, many allelochemicals extracted have been commercially applied to control weeds. Yet there is an immense prospect for allelochemicals to be used as a tool for new natural herbicide development.

## Limitations and future prospective

From the above review, it is clear that many allelopathic species have great potential for managing weeds. Regardless of recent advances in the research of allelopathy, there is still much more room to explore new allelochemicals and improve on the existing ones. We described the benefits and success of various allelopathic species in controlling weeds, but several problems have prevented a stronger interest in allelopathy use in conventional or organic agriculture.

Many allelochemicals are firstly very expensive to isolate and synthesize, regardless of having excellent herbicidal properties. One example is the cyclic tetrapeptide tentoxin, a good herbicide, but is very expensive to synthesize (Duke et al., 2002). Secondly, there is a misconception in the public that everything in nature is probably healthy. Several of the most toxic compounds known to humans, such as aflatoxin, fumonisins, and ricin are natural. AAL toxin and fumonisin are toxic to mammalian cells (Duke S. et al., 2000), while sorgoleone is reported to cause dermatitis (Inderjeet and Bhowmik, 2002). However, from an environmental toxicology perspective, the relatively short half-life of most allelochemicals in the field is desirable, but an herbicide must persist longer in the environment to get desired results (Ferguson et al., 2013). Additionally, the production and secretion of allelochemicals are highly influenced by plant age, temperature, light, soil, microflora, and nutritional status. While various plant species show great allelopathic potential, many of these species or their byproducts might not be suitable for use. In general, problems, such as cost, limited environmental stability, availability, and low herbicidal activity of many natural compounds have been the limiting factors in the research of allelopathy.

Regardless of many challenges in implementing the allelopathy concept for weed management, there is immense opportunity for exploring allelopathy as a new tool for weed management. Research lacks on the safety of using the known extracted allelochemicals on various crops. Future research needs to focus on the mechanisms of allelochemical selectivity, their modes of action, their interaction with different species, and ways to implement them. With modern technology in genetics, molecular biology, and biochemistry, we have the potential to learn about allelochemicals in much detail. Additionally, focusing on the development of transgenic allelopathy in crops through genetic engineering can provide a new way to implement the concept of allelopathy.

## Conclusion

The focus of this review was on different allelopathic species that release some forms of potent allelochemicals with the potential of being used in conventional or organic agriculture. The concept of allelopathy can be employed in the organic management of weeds and reduce our heavy reliance on synthetic herbicides. Although practices, such as intercropping, crop rotation, cover crops and mulching have been used conventionally for various benefits, integration of allelopathic crops would enhance its weed suppression benefit. Implementation of allelopathic plant extracts in combination with reduced doses of herbicides could be an alternative strategy for a sustainable weed management program. Thus far, there have been very few natural herbicides derived from allelochemical available in market, and there is a need for more with improved crop selectivity. With the modern biotechnological tools and improved extraction methods, more allelochemicals will be identified, tested, and used for weed management. Currently, it is difficult to replace chemical weed management entirely, but an integrated weed management approach may lead to success. These allelochemicals or the byproducts of allelopathic species can be integrated with other weed management practices to get better weed control, reduce herbicide use, reduce production costs, and avoid any herbicide resistance.

## Author contributions

YK conducted the literature searching, reviewed and wrote the manuscript with assistance of JC and SM. All authors contributed to the article and approved the submitted version.

## Conflict of interest

The authors declare that the work was conducted in the absence of any commercial or financial relationships that could be construed as a potential conflict of interest

## Publisher’s note

All claims expressed in this article are solely those of the authors and do not necessarily represent those of their affiliated organizations, or those of the publisher, the editors and the reviewers. Any product that may be evaluated in this article, or claim that may be made by its manufacturer, is not guaranteed or endorsed by the publisher.
